# P-357. Maximizing Value in Public Healthcare: Assessing the Financial Return on Investment in Surgical Infection Prevention at a Philanthropic Teaching Hospital Serving Exclusively Public Patients

**DOI:** 10.1093/ofid/ofae631.558

**Published:** 2025-01-29

**Authors:** Raquel Bandeira, José Américo Bahia Filho, Vespasiano C Luz Neto, Glauco Messias, Gabrielle R Mota, Thiago C Gontijo, Gabriel Colen, Ana Carolina Morganti, Ana Paula Ladeira, Lívia Miranda, Bráulio R G M Couto, Beatriz Messias, Isabela Messias

**Affiliations:** Hospital Metropolitana Doutor Célio de Castro, Belo Horizonte, Minas Gerais, Brazil; Hospital Universitário Ciências Médicas (HUCM), Belo Horizonte, Minas Gerais, Brazil; Hospital Universitário Ciências Médicas (HUCM), Belo Horizonte, Minas Gerais, Brazil; Hospital Universitário Ciências Médicas (HUCM), Belo Horizonte, Minas Gerais, Brazil; Hospital Universitário Ciências Médicas (HUCM), Belo Horizonte, Minas Gerais, Brazil; University Hospital Medical Sciences, Belo Horizonte, Minas Gerais, Brazil; Hospital Universitário Ciências Médicas (HUCM), Belo Horizonte, Minas Gerais, Brazil; Hospital Universitário Ciências Médicas (HUCM), Belo Horizonte, Minas Gerais, Brazil; Biobyte Tecnologia em Epidemiologia, Belo Horizonte, Minas Gerais, Brazil; Faculdade Dinâmica Vale do Piranga - FADIP, Viçosa, Minas Gerais, Brazil; AMECI – Associação Mineira de Epidemiologia e Controle de Infecções, Belo Horizonte, Minas Gerais, Brazil; Hospital Universitário Ciências Médicas (HUCM), Belo Horizonte, Minas Gerais, Brazil; Hospital Universitário Ciências Médicas (HUCM), Belo Horizonte, Minas Gerais, Brazil

## Abstract

**Background:**

This study aimed to evaluate the cost-effectiveness of hospitals’ continuous investments in HAI prevention, focusing specifically, on the prevention of SSIs. The objective was to estimate the impact of SSIs on hospital profitability.

**Figure 1:**
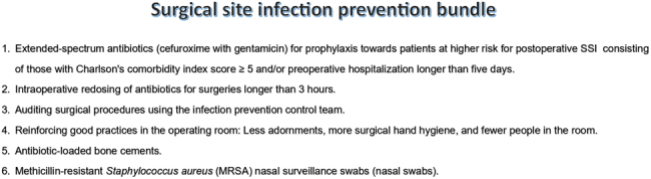
The infection prevention programs to prevent Surgical Site Infections (SSIs)

The infection prevention programs to prevent Surgical Site Infections (SSIs)

**Methods:**

A single-center retrospective cohort study was conducted between January 2019 and September 2023, involving patients undergoing arthroplasty, small bowel surgeries, cholecystectomy, herniorrhaphy, and open fracture reduction. The cost of each infection was obtained in the literature. The study compared SSI incidence between 2019-2022 and 2023. The hospital implemented a Value Management Office (VMO) in 2022 to accelerate the dissemination of value-based healthcare, with a focus on IPP, including measures to prevent SSIs. The infection prevention programs to prevent SSI consisted of maximum adherence to antimicrobial prophylaxis, SSI rate feedback for surgical team with root cause, auditing surgical procedures, reinforcing good practices in the operating room and improvement of the materials and sterilization center.

**Table 1:**
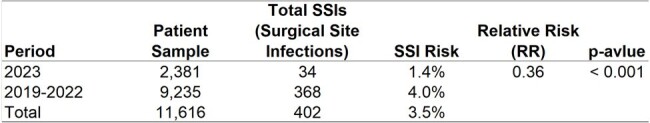
Comparison of Surgical Site Infection Rates: Baseline Period (2019-2022) vs. Current Period (2023) Comparison of Surgical Site Infection Rates: Baseline Period (2019-2022) vs. Current Period (2023)

**Results:**

During the baseline period, January 2019 to December 2022, 9,235 patients were included, being 59% female and a mean age of 51 years. 368 were diagnosed with SSI and the mortality rate was 1.4%. When an SSI occurs, the length of stay is longer (p=0.001) and the risk of death doubles (RR=2.1; p=0.033). The analysis shows a remarkable 64% decrease in SSI rates, from 4% in 2019-2022 to 1.4% in 2023. This translates into 63 prevented infections and 2 fewer deaths. The study attributes this success to implemented preventive measures because there was no significant difference in terms of the duration of the surgery (p= 0.411) and age of patients in each group (p=0.843). Additionally, the reduced SSI rate led to shorter hospital stays and an estimated monthly cost savings of US$52,160-US$93,865.

**Table 2:**
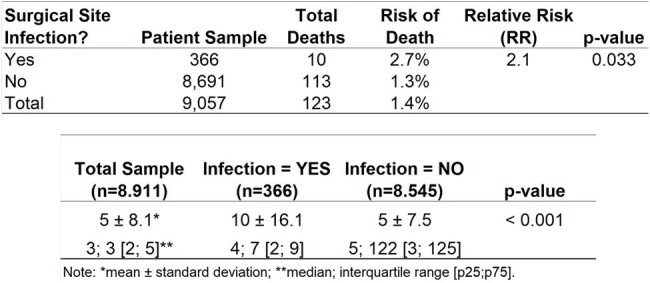
Impact of Surgical Site Infections on Mortality Risk and Extended Hospital Stay Impact of Surgical Site Infections on Mortality Risk and Extended Hospital Stay

**Conclusion:**

Investing in interventions aimed at reducing adverse events, such as SSIs, is essential for enhancing patient care and safety. This study highlights the significant financial returns associated with investing in infection prevention: 52,160 to 93,865 USD, particularly in reducing surgical infections.

**Table 3:**
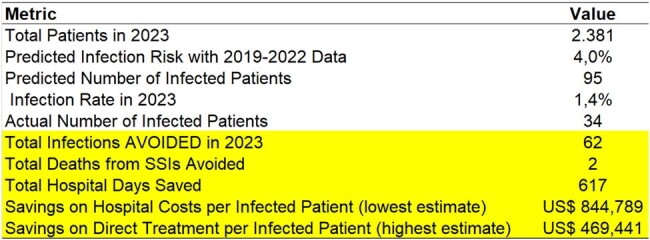
Reduction in Costs, Hospital Stay Duration, and Prevented Deaths Associated with Surgical Site Infections Reduction in Costs, Hospital Stay Duration, and Prevented Deaths Associated with Surgical Site Infections

**Disclosures:**

**All Authors**: No reported disclosures

